# Quantitative Comparison of SPECT and PET Performance for Clinical Theranostic Applications

**DOI:** 10.2967/jnumed.125.270987

**Published:** 2026-03

**Authors:** Isabella Salerno, Nicholas Dunn, Haley White, Wilnellys Miyazaki, Kyle Jeziorski, Delynn Silvestros, Abhinav Jha, Richard Laforest, Mercy I. Akerele, M. Allan Thomas, Daniel L.J. Thorek

**Affiliations:** 1 Medical Physics Division, Department of Radiation Oncology, Washington University in St. Louis School of Medicine, St. Louis, Missouri;; 2 Program in Quantitative Molecular Therapeutics, Washington University in St. Louis School of Medicine, St. Louis, Missouri;; 3 Division of Nuclear Medicine, Department of Radiology, Washington University in St. Louis School of Medicine, St. Louis, Missouri;; 4 Precision Radiotheranostics Translation Center, Mallinckrodt Institute of Radiology, Washington University in St. Louis School of Medicine, St. Louis, Missouri;; 5 Oncologic Imaging Program, Siteman Cancer Center, Washington University in St. Louis School of Medicine, St. Louis, Missouri;; 6 Department of Biomedical Engineering, Washington University in St. Louis, St. Louis, Missouri; and; 7 Division of Interventional Radiology, Department of Radiology, Washington University in St. Louis School of Medicine, St. Louis, Missouri

**Keywords:** SPECT, PET, quantitative imaging, theranostics, image performance

## Abstract

Radiopharmaceutical therapy is an emerging approach to treat metastatic disease, with the potential to enable personalized care through imaging. With the growing interest in quantitative imaging, there remains a need for systematic assessment of imageable radionuclides and scanner performance under comparable conditions. This motivated us to quantitatively and qualitatively compare SPECT and PET performance using standardized evaluation procedures to assess their effectiveness in imaging applications for targeted radiotherapy. **Methods:** The National Electrical Manufacturers Association International Electrotechnical Commission body phantom was used to assess recovery coefficients, contrast, and image quality, including spatial resolution and noise. SPECT performance was evaluated using ^99m^Tc (a low-energy γ-ray–emitting isotope), ^203^Pb (a surrogate for the therapeutic α-particle–emitting ^212^Pb), and theranostic ^177^Lu. PET performance was tested using ^18^F, ^89^Zr, and ^64^Cu as imaging surrogates. **Results**: PET demonstrated superior spatial resolution and accurate activity recovery compared with SPECT, which was limited by lower sensitivity, photon scatter, and collimator design constraints. Among PET isotopes, ^18^F showed consistently high quantitative accuracy, whereas ^89^Zr and ^64^Cu performed similarly, with only minor reductions in performance metrics. For SPECT, ^99m^Tc outperformed ^203^Pb and ^177^Lu in both lesion detectability and activity recovery. ^203^Pb and ^177^Lu showed poor quantitative accuracy; however, increasing iterations in reconstruction improved results. **Conclusion:** These findings underscore the importance of selecting appropriate imaging modalities, isotopes, and reconstruction parameters for theranostic applications. Limitations in quantitative accuracy must be addressed and acknowledged in the search for precise and effective treatment strategies. This study also demonstrates that ^203^Pb may serve as a suitable diagnostic partner to ^212^Pb and validates the use of these quantitative methodologies for the evaluation of PET and SPECT imaging tasks.

## Visual Abstract

**Figure:**
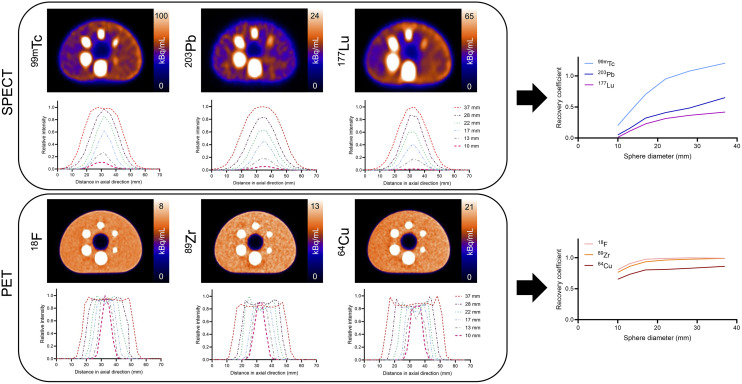

Theranostics is a rapidly evolving field that offers an innovative approach to cancer diagnosis and treatment. Uniquely, this paradigm provides the ability to monitor and quantitate the distribution of a therapeutic, enabling personalized patient treatment. In some applications, a single radioisotope can serve both diagnostic and therapeutic purposes. Alternatively, theranostic pairs may be used, whereby analogous agents are applied for imaging and treatment (1*–*3) to enable the precise visualization of therapeutic radioisotope distribution, even when the therapeutic agent itself is not directly imageable.

SPECT and PET are key imaging technologies that are instrumental in providing insights into both molecular and functional processes (4). Although SPECT can be a quantitative tool, it primarily has been used to measure relative radiopharmaceutical uptake rather than absolute quantitation (5). Despite advancements, such as the incorporation of CT-based corrections to improve accuracy (6), SPECT is still widely regarded as inferior to PET. ^99m^Tc remains the most widely used isotope in SPECT imaging, valued for its distinct 140-keV γ-emission and versatility in diagnostic applications (7,8). Imaging using the 208-keV γ-emission of therapeutic ^177^Lu has become commonplace after recent approvals (9,10). There is also increasing interest in ^203^Pb, which has the potential to be a surrogate for the therapeutic ^212^Pb (11*–*14). ^212^Pb decays by β-emission to ^212^Bi, followed by α-emissions along the decay chain until it reaches stable ^208^Pb (11,15). It is being evaluated for targeted α-therapy in several indications (16*–*18). A diagnostic counterpart would be favorable because of the difficulties that arise with the mixture of high-energy γ-emissions from ^212^Pb progeny, which reduce image quality (14,15). ^203^Pb has a unique ability to circumvent these issues, as the chemical properties of both isotopes are identical, making this pair suitable to be labeled to the same compound for diagnostic and therapeutic radiopharmaceuticals.

PET demonstrates greater sensitivity and superior quantitative accuracy, which make it indispensable for both detection and patient management applications. Among PET isotopes, ^18^F is the most commonly used PET radionuclide because of its high positron yield and relatively low positron energy (19). ^64^Cu is a longer-lived PET isotope that allows for the evaluation of small-molecule pharmacokinetics over extended periods and delayed lesion imaging (20) and is a putative surrogate for therapeutic electron-emitting ^67^Cu. Longer-lived isotopes, such as ^89^Zr, are also emerging in antibody-based imaging (21,22). Both ^89^Zr and ^64^Cu have higher positron energies and decreased positron fractions (23% and 17%, respectively) compared with ^18^F (19,20), which can contribute to their relatively reduced image quality (23).

PET systems are routinely evaluated using standardized National Electrical Manufacturers Association (NEMA) NU2 protocols, which emphasize quantitative performance through metrics such as spatial resolution, sensitivity, timing resolution, count errors, and image quality (24,25). In contrast, SPECT systems have historically been characterized using NEMA NU1 standards, which focus on relative performance (26). NU1 primarily specifies testing with ^99m^Tc at 140 keV. This lack of harmonization between PET and SPECT standards complicates efforts to validate and compare quantitative performance in theranostics. With modality-specific limitations, such as resolution degradation, collimator-dependent performance in SPECT, and positron range variation in PET, there remains a need for systematic assessment of isotope and equipment performance under comparable conditions.

This study aims to assess the qualitative and quantitative performance of both SPECT and PET using standard evaluation procedures with isotopes of interest in this emerging space. We were inspired by the work of Ryu et al. (27), who proposed the use of NEMA NU2 standards for PET and SPECT. We assessed image quality, spatial resolution, contrast, and activity recovery using multiple isotopes chosen for their wide variety of physical properties (Supplemental Table 1, available at http://jnm.snmjournals.org). Our work demonstrates the presence of both modality and isotope-specific considerations, and the continuing need for an appreciation of imaging medical physics considerations in the context of translational theranostics.

## MATERIALS AND METHODS

PET radionuclides were sourced from the cyclotron facility and nuclear pharmacy of the Mallinckrodt Institute of Radiology as dilute acidic solutions. Clinical-grade ^99m^Tc-sodium pertechnetate and ^177^Lu vipivotide tetraxetan were purchased from commercial sources. ^203^Pb chloride was purchased from the Department of Energy National Isotope Development Center and was produced at the University of Alabama Birmingham. All imaging systems used are in routine clinical use and maintained by technical staff accredited by the American College of Radiology.

### SPECT Configuration and Acquisition

SPECT images were acquired using a Symbia T6 Series SPECT/CT system (Siemens Healthineers), unless otherwise indicated. ^99m^Tc required the use of low-energy, high-resolution collimators, whereas ^203^Pb and ^177^Lu required the use of a medium-energy, low-penetration collimator. The main energy peak for ^99m^Tc was set at 140 keV, with a 15% energy window. An energy window centered on 279 keV was used for the main peak of ^203^Pb, with a 20% energy window. ^177^Lu was centered on photopeak 208 keV, with a 20% energy window.

### PET Configuration and Acquisition

PET scans were acquired on a Biograph Vision PET/CT system (Siemens Healthineers). The energy window of the PET scanner was the default 435–585 keV.

### NEMA International Electrotechnical Commission (IEC) Phantom Scanning

A NEMA IEC body phantom (Data Spectrum Corp.) was used to simulate a human torso in both SPECT/CT and PET/CT with hot spheres filled with activity dissolved in pure water, with either an approximate 10:1 diluted background (warm background) or no activity (cold background, water) as indicated. Standard sphere diameters were 37, 28, 22, 17, 13, and 10 mm, and activities for each were directly measured in a CRC-15R dose calibrator (Capintec) before imaging.

For SPECT/CT acquisitions, ^99m^Tc-, ^203^Pb-, and ^177^Lu-filled phantoms were scanned using 27-s steps across 64 views (128 projection images) in a body-contour orbit. All SPECT acquisitions used an 180° scan arc per detector head. Sphere concentration for the ^99m^Tc-filled phantom was 236 kBq/mL, and the background concentration was 23.7 kBq/mL. ^203^Pb sphere and background activity concentrations were 88.2 and 8.7 kBq/mL, respectively. ^177^Lu sphere and background activity concentrations were 661.2 and 64.9 kBq/mL, respectively. A higher activity concentration was selected to compensate for the lower yield of γ-emissions. A 3-dimensional iterative ordered-subset expectation maximization algorithm was used with resolution recovery, attenuation correction, and scatter correction. Reconstructions were performed with varying numbers of iterations (1, 2, 4, 8, 16, and 32) and 4 subsets, followed by a gaussian postprocessing filter with an 8.40-mm kernel. A low-dose CT scan was performed after all SPECT scans (130 kVp, 17 mAs, and 0.8 pitch).

Reconstruction parameters were based on our clinic’s protocol for ^99m^Tc SPECT imaging. ^203^Pb does not have defined reconstruction settings within our clinical software; however, prior studies have reported similar reconstruction settings, typically using 1–8 subsets and 10–30 iterations, followed by gaussian postfiltering with 9- to 12-mm kernels (14,15). Our clinical protocol for posttherapy ^177^Lu imaging includes reconstruction using 5 iterations, 10 subsets, and an 8-mm gaussian postprocessing filter. For this comparative study, subsequent spatial resolution, line profiles, and contrast measurements were evaluated with a reconstruction protocol (8 iterations, 4 subsets) and an 8.4-mm gaussian postprocessing filter. This was identified as the most balanced and representative protocol, offering sufficient contrast recovery with manageable noise levels while allowing us to maintain consistency across isotopes and reflect clinical reconstruction conditions.

An additional ^177^Lu-filled NEMA phantom was scanned on an Optima NM/CT 640 (GE HealthCare). Acquisition parameters were the same used with the Symbia, except for the number of views (120 projection images) (supplemental materials).

PET/CT scans were performed, with a total acquisition time of 1,805 s. All acquisitions were reconstructed using a method that combined point-spread function modeling and time-of-flight information, with 4 iterations and 5 subsets. Attenuation and scatter correction were applied using a model-based approach with relative scatter scaling. Sphere and warm-background activities were 66.3 and 5.9 kBq/mL, respectively, for ^18^F; 102.4 and 10.2 kBq/mL, respectively, for ^89^Zr; and 149.1 and 15.5 kBq, respectively, for ^64^Cu. Low-dose CT scans were performed after all PET scans (120 kVp, 25 mAs, and 0.8 pitch).

### Analyses

MIM Software version 7.4.2 was used to measure activity concentration from volumes of interest (VOIs) of known dimensions. We used CT to define the actual sphere volumes. VOIs were defined for each sphere in the NEMA phantom, and the background VOI was determined from the full body contour, excluding the sphere VOIs. Recovery coefficients (RCs) for the hot spheres were calculated from the activity measured by reconstructed SPECT and PET scans and compared with the true activity values measured with the dose calibrator. To convert SPECT image counts to activity concentrations, a quantitative factor was applied, using the method described by Kupitz et al. (28):

$$\mathrm{QF}=\;\frac{1}{C_{s}\;\times \;\Delta t\;\times \;V_{\mathrm{voxel}}},$$ Eq. 1

where $C_{s}$ is the camera sensitivity taken from the system calibration provided by the SPECT manufacturer, Δt is the total acquisition time, and $V_{\mathrm{voxel}}$ is the volume of a single reconstructed voxel.

Image contrast values were derived from reconstructed warm-background NEMA phantom scans and normalized to the known hot sphere–to-background ratio for each isotope using the following contrast (*C*) equation:

$$C=\;\frac{\left(\frac{{\mathrm{VOI}}_{m}}{{\mathrm{VOI}}_{b}}\right)}{A},$$ Eq. 2

where ${\mathrm{VOI}}_{m}$ represents the mean image-derived activity within a hot-sphere VOI, and ${\mathrm{VOI}}_{\text{b}}$ represents the mean image-derived activity in the background region. The ratio $\frac{{\mathrm{VOI}}_{m}}{{\mathrm{VOI}}_{b}}$ corresponds to the measured sphere-to-background activity ratio for the reconstructed images, normalized by the known true activity ratio, *A*.

## RESULTS

### SPECT Reconstruction and Recovery

**FIGURE 1.**
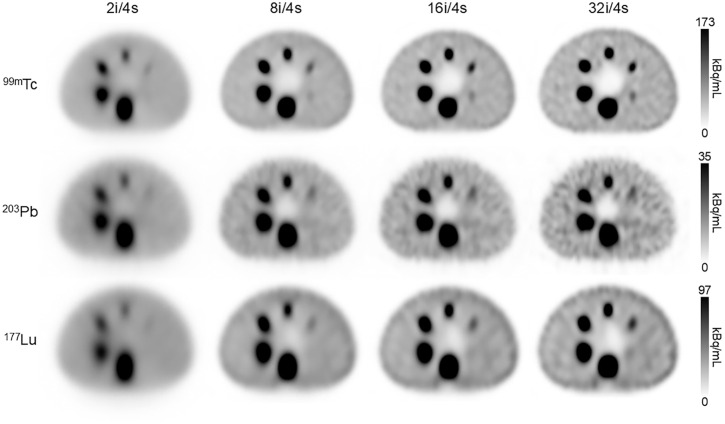
SPECT warm-background NEMA phantom scans with reconstructions of ^99m^Tc, ^203^Pb, and ^177^Lu using different iterations and fixed number of subsets. Reconstruction was performed with 2i/4s, 8i/4s, 16i/4s, and 32i/4s (left to right). i = iterations; s = subsets.

We began by evaluating SPECT instrument performance for ^99m^Tc, ^203^Pb, and ^177^Lu using the NEMA IEC body phantom with warm background. Figure 1 displays reconstructions at 4 iteration levels with 4 subsets. As expected, increasing the number of iterations improved image quality, but only to an extent. On visual inspection, detectability of all spheres was lowest at 2 iterations (Fig. 1). The smallest sphere (10 mm) in the ^99m^Tc scans became visible only after 8 iterations, but it remained undetectable in the ^203^Pb and ^177^Lu scans at all iteration levels. The greatest discernability for all spheres was observed with the reconstruction setting of 32 iterations and 4 subsets; however, increased iterations introduced background noise and hyperintensity at phantom edges.

**FIGURE 2.**
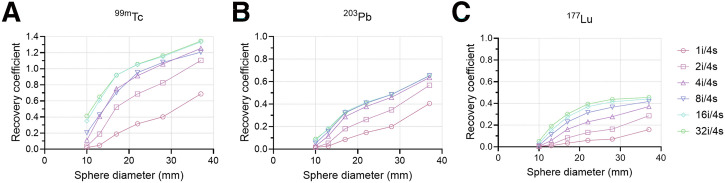
RCs of reconstructed SPECT scans for ^99m^Tc (A), ^203^Pb (B), and ^177^Lu (C). Each reconstruction was performed with 4 subsets and varying number of iterations (1–32). i = iterations; s = subsets.

Figure 2 shows the RCs for 6 different SPECT reconstruction settings for ^99m^Tc, ^203^Pb, and ^177^Lu. RCs increased with the number of iterations across all sphere sizes, with the most significant improvements observed after reaching 4 and 8 iterations, after which increases in iterations provided minimal additional benefit, indicating convergence of the reconstruction algorithm. We observed RC values above 1.0 for the largest ^99m^Tc spheres, most likely attributable to resolution recovery in the reconstruction algorithm.

### Spatial Resolution

**FIGURE 3.**
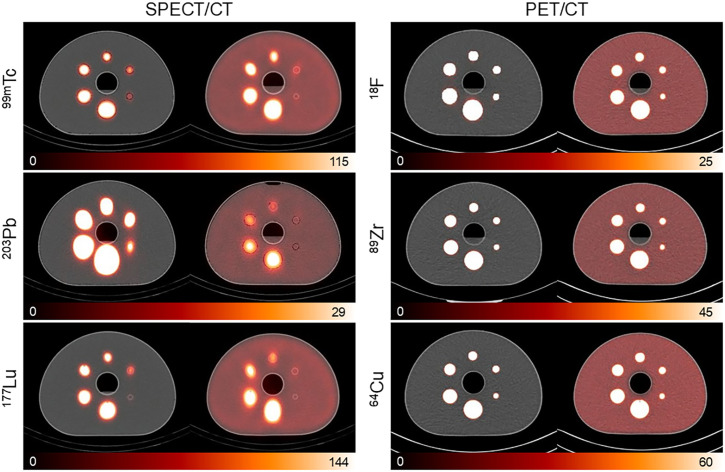
NEMA body phantom scans for SPECT and PET isotopes. In each panel, CT-fusion images include cold- and warm-background (10:1 activity concentration) scans displayed on left and right, respectively. Scale bars are expressed in kBq/mL.

Cold- and warm-background scans for both SPECT/CT and PET/CT are presented in Figure 3. The reconstructed images highlight the disparity in image quality between modalities. Notably, the 2 smallest spheres were indistinguishable from the background activity in the warm-background scans of ^203^Pb and ^177^Lu SPECT.

**FIGURE 4.**
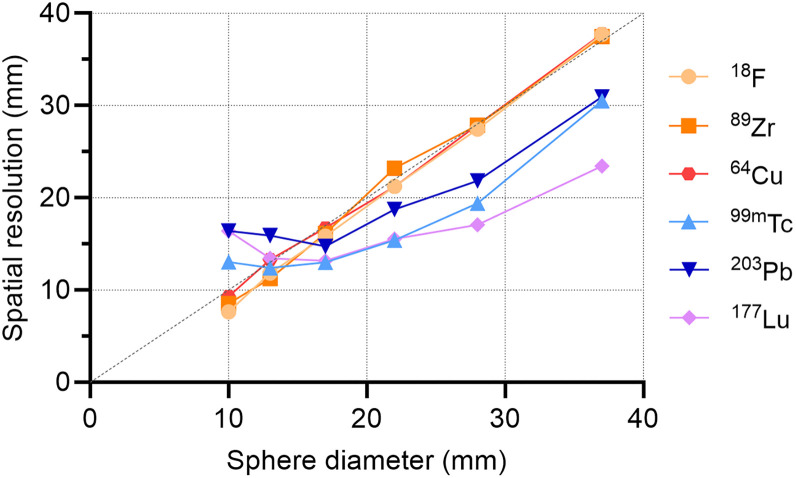
Spatial resolution as function of sphere diameter for ^18^F, ^89^Zr, and ^64^Cu by PET and ^99m^Tc, ^203^Pb, and ^177^Lu by SPECT for fixed activity concentrations.

Spatial resolution performance was evaluated by examining line profiles across the center of the filled spheres of cold-background scans. The equivalent dashed line in Figure 4 demonstrates the ideal relationship between spatial resolution and sphere diameter. PET acquisitions followed the expected relationship, while SPECT systems deviated across the sphere diameters. As seen in the 3 smallest spheres, the SPECT imaging system had an intrinsic resolution that imposed a limit on the ability to further improve resolution beyond a certain point, regardless of sphere size.

**FIGURE 5.**
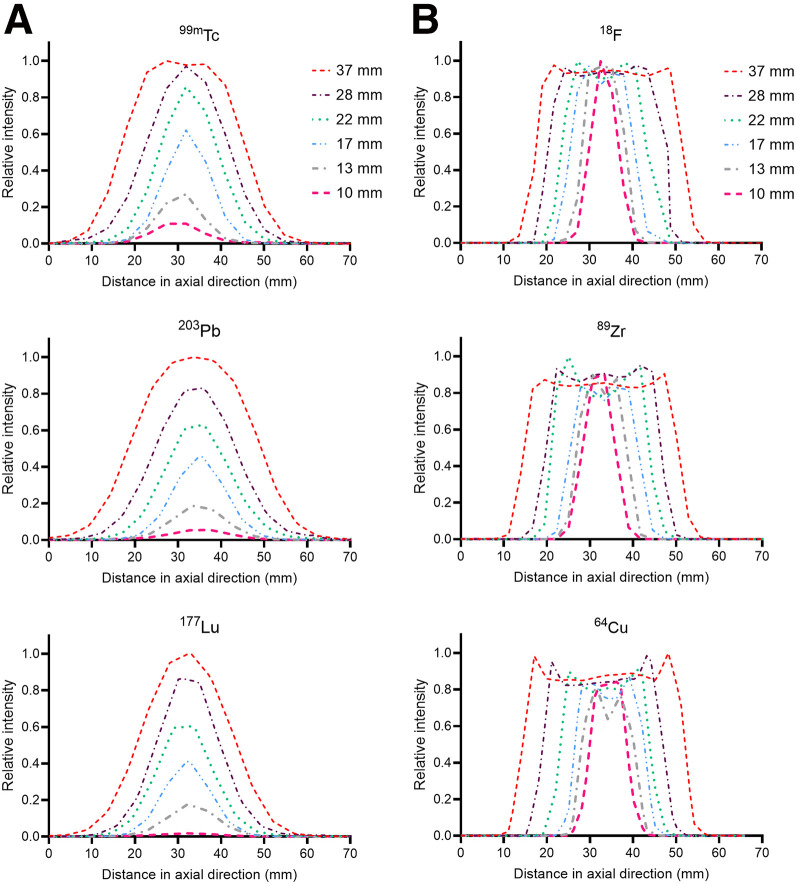
Line profiles through center of NEMA phantom spheres of varying diameters as measured using SPECT (A) and PET (B) isotopes.

Line profiles were delineated from the SPECT and PET cold-background scans for all spheres, as depicted in Figure 5. The superior resolution and sensitivity of PET produced more consistent and distinct line profiles, accurately reflecting the homogeneous activity concentration within spheres. Conversely, the coarser resolution and sensitivity of SPECT resulted in a gradual decline in relative intensity as sphere diameter decreased. These factors were attributable to the partial-volume effects in SPECT reconstruction.

### Activity and Contrast Recovery

**FIGURE 6.**
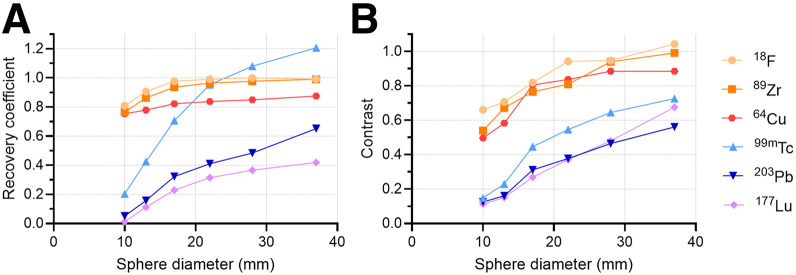
(A) RCs for ^18^F PET, ^89^Zr PET, ^64^Cu PET, ^99m^Tc SPECT, ^203^Pb SPECT, and ^177^Lu SPECT (8 iterations/4 subsets SPECT reconstruction). (B) Image contrast for SPECT and PET isotopes across varying sphere diameters. Contrast values were derived from warm-background NEMA phantom scans with 10:1 hot-sphere–to-background ratio.

Figure 6A depicts the RCs for hot spheres in the NEMA phantom for SPECT and PET acquisitions. As expected, ^18^F PET exhibited the most accurate activity recovery, with the smallest sphere achieving 80.9% recovery, compared with only 20.3% for ^99m^Tc SPECT. Overall, PET isotopes displayed a consistent trend, with recovery remaining above 80% except for ^89^Zr in the smallest sphere (10 mm) and ^64^Cu in the 13- and 10-mm spheres. In contrast, SPECT isotopes showed lower recovery rates, which were dependent on sphere diameter. The 2 smallest sphere diameters for all SPECT isotopes were poorly quantified.

Image contrast was also evaluated across spheres for each isotope (Fig. 6B). Spheres for ^203^Pb and ^177^Lu (the 2 smallest spheres) and ^99m^Tc (the smallest sphere) had contrast values below 0.16. These quantitative findings are consistent with the visual observations shown in Figures 1 and 3, in which the 10-mm sphere was not visually detectable in warm-background ^203^Pb and ^177^Lu scans and only marginally detectable in warm-background ^99m^Tc scans.

### Scanner Considerations

There is a wide range of vendor hardware and instrument configurations for theranostic imaging. Testing across the continuum of these many systems is beyond the scope of this study; however, we performed imaging of a SPECT (^177^Lu) and PET (^64^Cu) emitter in the standard phantom across multiple systems to illustrate the impact of this important consideration in a controlled setting. These data showed the variability in quantitative image measures across vendors for SPECT (Supplemental Figs. 1–3) and distinct installations of identical hardware for PET (Supplemental Fig. 4).

## DISCUSSION

SPECT and PET are crucial imaging modalities in nuclear medicine with central roles in the expanding theranostics landscape. We assessed the performance of these systems through a comprehensive analysis of image quality, spatial resolution, activity recovery, and image contrast for 6 highly relevant diagnostic isotopes, providing insight into their capabilities and limitations in theranostic applications.

Our data reiterate that quantitative SPECT imaging is complicated by factors such as lower sensitivity, increased partial-volume effects, and the need for complex postimaging corrections relative to PET (29,30). Our findings show that reconstruction parameters, particularly the number of iterations, significantly influence image contrast and quantitative accuracy, which are further complicated by isotope-specific characteristics. Particularly in decreased iterations, we observed distortions in spheres (appearing elongated rather than circular in cross-section), which is a well-established phenomenon in SPECT. This was observed for all SPECT isotopes and can be attributed to reduced count sensitivity and angular sampling. Increasing iterations improved this artifact and image contrast; however, noise also increased, and gains in recovery plateaued beyond a certain point.

Even with refined reconstruction, system-dependent factors, such as detector response and collimator design, shaped performance. Notably, the smallest sphere was poorly resolved under cold-background conditions for ^203^Pb and ^177^Lu, and detection was further challenged with warm-background conditions for all SPECT scans (Figs. 1, 3, 5, and 6).

As expected, ^99m^Tc and ^18^F performed well with their respective imaging modalities. Overall, ^18^F PET demonstrated the highest performance, offering superior spatial resolution and the most accurate quantitative evaluation. ^99m^Tc SPECT provided strong image contrast and lesion detectability under most conditions. In addition, we evaluated ^203^Pb, ^64^Cu, and ^89^Zr to investigate the imaging performance of longer-lived isotopes that serve as surrogates for therapeutic agents. ^64^Cu and ^89^Zr performed comparably to ^18^F, with observable degradation of spatial resolution, contrast, and activity recovery. This was the expected outcome, given the differences in positron energy and additional emissions (Supplemental Table 1) (19).

^203^Pb was specifically used to test its potentiality as an imaging counterpart to the therapeutic α-emitting ^212^Pb. ^203^Pb has a much higher energy γ-emission (279 keV) than ^99m^Tc, making it prone to septal penetration and Compton scattering. However, it did demonstrate strong imaging performance, with only slightly lower spatial resolution and image contrast. Quantitative accuracy remained a challenge, as the largest sphere diameter yielded an RC of only 65.2%. Recovery declined sharply with decreasing sphere size, reaching just 5.2% for the smallest sphere. Despite reduced quantification, the image quality of ^203^Pb SPECT in Figures 1 and 3 is comparable to that of ^99m^Tc SPECT, particularly with increased reconstruction iterations.

^177^Lu and ^64^Cu (an isotopologue of ^67^Cu) were also evaluated for their combined diagnostic and therapeutic potential. ^64^Cu quantitation trailed that of ^18^F and ^89^Zr, attributable in part to its low positron yield. In addition, ^64^Cu demonstrated edge-related effects (Gibbs artifacts) in line profiles (Fig. 5). The relative intensity increase at the sphere boundaries is particularly noticeable in the 3 largest spheres. All PET images were reconstructed with point-spread function modeling, which can contribute to this phenomenon (31). ^177^Lu was outperformed, both qualitatively and quantitatively, by the other SPECT isotopes investigated. Increasing the number of reconstruction iterations did improve image quality but resulted in hyperintensity at the NEMA phantom boundary and an attenuation correction artifact at the inferior edge of the largest sphere (Fig. 1). Recovery remained poor, with the largest sphere reaching only 45% even at 32 iterations and 4 subsets.

These results highlight a key challenge in quantitative SPECT—even with a well-characterized isotope, such as ^99m^Tc, quantification is not ideal. Activity concentrations can be overestimated for larger volumes and significantly underestimated for objects smaller than the system’s spatial resolution. This underestimation is largely attributed to partial-volume effects, which cause the spill out of counts from small, high-uptake regions into adjacent areas, thereby increasing the absolute bias (30). Overestimation, on the other hand, can result from factors such as photon scatter within the medium or certain iterative reconstruction parameters. The differences between measured and true activities are exacerbated in the context of ^177^Lu and ^203^Pb, which challenge the precision of dosimetry efforts. We observed these effects in RCs as well as line-profile performance through NEMA spheres (Fig. 5). The maximum relative intensity differed by 89%, 95%, and 98% for the largest (37 mm) and smallest (10 mm) diameter spheres for ^99m^Tc, ^203^Pb, and ^177^Lu, respectively. Object size and volume were the most significant contributors to this discrepancy. The maximum difference observed across all relative intensity values for ^18^F, ^89^Zr, and ^64^Cu were 3%, 16%, and 20%, respectively. These results emphasize the importance of prioritizing PET diagnostic surrogates when possible.

It is important to note that we can thoroughly assess quantitative SPECT using standardized methods similar to those used in PET quality assurance. Implementing the approaches described in this study will allow for more consistent system comparisons across and within institutions. This is particularly relevant in cancer theranostics, as accurate and reliable dosimetry depends on consistent quality assurance.

This study had several limitations. Quantitative SPECT can be approached in many ways; in our case, we applied a calibration factor to convert counts to units of activity. Alternative calibration and quantification methods described throughout the literature may yield different results. For concision, we evaluated a single iterative reconstruction method. As discussed, reconstruction parameters can substantially influence both quantitative and qualitative outcomes, and alternative quantitation methods may be warranted (32,33). It is also important to acknowledge that this was a phantom-based study, which does not fully reflect the complexity of clinical imaging.

Together, these data reinforce the importance of in-depth evaluation of quantitative performance in nuclear medicine imaging. As we have demonstrated, there are significant uncertainties that arise, even under controlled imaging experiments. Although further work is needed to systematically incorporate and account for SPECT-related uncertainties and differences in imaging system configurations, our results show that ^203^Pb can achieve image quality comparable to that of conventional SPECT isotopes and that ^177^Lu quantitation must be carefully considered. Furthermore, the methodologies presented in this study offer a comprehensive framework for evaluating both SPECT and PET performance, contributing to the advancement of quantitative imaging in theranostics.

## CONCLUSION

PET demonstrated superior spatial resolution and accurate activity recovery compared with SPECT, which was limited by lower sensitivity, photon scatter, and collimator design constraints. As SPECT continues to evolve from a qualitative to quantitative imaging tool, careful consideration of system-dependent factors and quantification uncertainties must be recognized. Incorporating this framework into future analysis can improve qualitative and quantitative accuracy, allowing for greater confidence in treatment planning, uptake estimation, and dosimetry.

## DISCLOSURE

This work was supported by National Institutes of Health grants R01CA240711, R01CA229893, R01EB031962, and R01EB031962-03S1. No other potential conflict of interest relevant to this article was reported.

## Notes

KEY POINTS **QUESTION:** How do SPECT and PET compare in terms of quantitative accuracy and overall system performance when evaluated with theranostic isotopes under standardized testing procedures? **PERTINENT FINDINGS:** SPECT isotopes (^99m^Tc, ^203^Pb, and ^177^Lu) exhibited greater variability in all assessed areas compared with PET isotopes (^18^F, ^89^Zr, and ^64^Cu), even under standardized evaluation conditions. These findings highlight the need for systematic isotope- and modality-specific validation to support reliable quantitative applications in theranostics. **IMPLICATIONS FOR PATIENT CARE:** Incorporating a framework that accounts for the limitations and uncertainties of nuclear imaging can enhance quantitative accuracy, enabling greater confidence in treatment planning, uptake estimation, and dosimetry.
